# A Review on Mechanistic Insight of Plant Derived Anticancer Bioactive Phytocompounds and Their Structure Activity Relationship

**DOI:** 10.3390/molecules27093036

**Published:** 2022-05-09

**Authors:** Kishor Mazumder, Asma Aktar, Priyanka Roy, Biswajit Biswas, Md. Emran Hossain, Kishore Kumar Sarkar, Sitesh Chandra Bachar, Firoj Ahmed, A. S. M. Monjur-Al-Hossain, Koichi Fukase

**Affiliations:** 1 Department of Pharmacy, Jashore University of Science and Technology, Jashore 7408, Bangladesh; asmaaktar121039@gmail.com (A.A.); priyankaroy.pharmacy@gmail.com (P.R.); bb@just.edu.bd (B.B.); emran.du2011@gmail.com (M.E.H.); kishorekumar0811@gmail.com (K.K.S.); 2 School of Optometry and Vision Science, UNSW Medicine, University of New South Wales (UNSW), Sydney, NSW 2052, Australia; 3 Department of Pharmacy, Faculty of Pharmacy, University of Dhaka, Dhaka 1207, Bangladesh; bacharsc@du.ac.bd (S.C.B.); firoj72@du.ac.bd (F.A.); 4 Department of Pharmaceutical Technology, Faculty of Pharmacy, University of Dhaka, Dhaka 1207, Bangladesh; monjur.shiplu@du.ac.bd; 5 Department of Chemistry, Graduate School of Science, Osaka University, 1-1 Machikaneyama, Toyonaka, Osaka 560-0043, Japan

**Keywords:** neoplastic disease, proliferation, anticancer bioactive phytocompounds, structure activity relationship (SAR), cytotoxic agents

## Abstract

Cancer is a disorder that rigorously affects the human population worldwide. There is a steady demand for new remedies to both treat and prevent this life-threatening sickness due to toxicities, drug resistance and therapeutic failures in current conventional therapies. Researchers around the world are drawing their attention towards compounds of natural origin. For decades, human beings have been using the flora of the world as a source of cancer chemotherapeutic agents. Currently, clinically approved anticancer compounds are vincristine, vinblastine, taxanes, and podophyllotoxin, all of which come from natural sources. With the triumph of these compounds that have been developed into staple drug products for most cancer therapies, new technologies are now appearing to search for novel biomolecules with anticancer activities. Ellipticine, camptothecin, combretastatin, curcumin, homoharringtonine and others are plant derived bioactive phytocompounds with potential anticancer properties. Researchers have improved the field further through the use of advanced analytical chemistry and computational tools of analysis. The investigation of new strategies for administration such as nanotechnology may enable the development of the phytocompounds as drug products. These technologies have enhanced the anticancer potential of plant-derived drugs with the aim of site-directed drug delivery, enhanced bioavailability, and reduced toxicity. This review discusses mechanistic insights into anticancer compounds of natural origins and their structural activity relationships that make them targets for anticancer treatments.

## 1. Introduction

Plants are promising assets for the discovery and development of new therapies and their use can be traced back to ancient times. Present day figures estimate that there are about 250,000 species of flowering plants in the world and of those, 155,000 are situated in the tropics [1]. Bioactive plant-derived phytocompounds can be anticipated to play a more and more substantial function in the development of new drugs. Among the large species of plants, a few number of there have been explored for assaying biological activities as well as screening bioactive phytocompounds. Systematic research of the undiscovered plant source can be commercially profitable resource to obtain novel biomolecules with desired pharmacological activities [2]. Hence, with this massive reservoir, a high quality and rational approach is the demand of this era for finding unused drugs with anticancer potential. 

Cancer is an extreme metabolic disorder that has seen significant advancement in treatment plans and preventative remedies. It is also called neoplastic disease, characterized by the uncontrolled proliferation followed by the constant multiplication of human cells. This leads to the development of tumors of harmful malignant cells with the capacity to be metastatic [3]. Cancer is initiated by mutations of following genes; (a) oncogenes: RAS, Bcl-2, RAF, and MYC; (b) tumor suppressor genes: NF1, NF2, p53; and (c) DNA repair genes: p21, p22, p27, p51, p53, and tool box for DNA. These resultant genetic modifications can be triggered by an imbalance in hormones and in the immune system, and by exposure to external stimuli (e.g., radiation, pesticide, tobacco smoking, carcinogenic chemicals and metals, aflatoxin and several steroidal medications) [4]. A number of treatment approaches, such as chemotherapy, radiation therapy, thermal ablation, and resection have been developed as anticancer therapies. The success rate of these therapies is diminished by toxicities, drug resistance, recurrence and treatment failure. Moreover, the cost of these therapies is out of reach for lower income populations [5]. Thus, the current research has aimed to discovery of natural biomolecules with potent anticancer activity as well as negligible side effects with a view to avail them from laboratory to bedside of patients at lower cost. 

Medicinal plants, through the diversity of their chemical constituents, are vital for the invention of novel substances with effectiveness against tumors and other malignant cells. Drug development and discovery from medicinal plant-derived secondary metabolites has become an essential part of searching for anticancer therapies over the centuries. Current screenings of medicinal plants for bioactive constituents with effectiveness as an anticancer agent have supplied modern medicine with compelling cytotoxic drugs: for example, vinca alkaloids, podophyllotoxins and taxanes [6]. Over 60% of the commercially accepted anticancer agents inside the US (from 1983 to 1994) originated from natural sources [7,8]. About 771 of new cytotoxic agents were listed to be in the pipeline in 2014, and since 2011, the Food and Drug Administration (FDA) approved 55 new drugs [9]. The most well-known plant-derived anticancer compounds of medical importance include those especially good at attacking the cytoskeleton system of cell microtubules which include the vincristine, vinblastine, and taxanes, e.g., docetaxel (Taxotere), paclitaxel (Taxol) and others [10,11,12]. 

From extraction to isolation, characterization and formulation in the drug development process, the major critical and vital steps are the analysis of structural features and the structure activity relationship (SAR), as well as the exploration of mechanistic insights. In consideration of the development of phytocompounds as effective anticancer drug products, they should be proven as clinically safe and therapeutically effective in different phases of clinical trials. Data regarding SAR analysis and mechanism of activity are considered as key factors of safety and effectiveness in laboratory research which further accelerates the research to clinical trials. In this review, the authors attempted to discuss the profound bioactive compounds with anticancer activity with a view to mechanistic insight, as well as SAR. 

## 2. Method

### 2.1. Search Scheme

Renowned scientific databases such as PubMed (https://pubmed.ncbi.nlm.nih.gov/), Google scholar (https://scholar.google.com/), Springer Link (http://link.springer.com/), ScienceDirect (http://www.sciencedirect.com/), Scopus (http://www.scopus.com/), and Wiley Online Library (http://onlinelibrary.wiley.com/) (all accessed on 10 September 2021) to search literatures by emphasizing specific terminologies, such as “anticancer”, “medicinal plants”, “bioactive compounds”, “structure activity relationship”, and “anticancer mechanism”. Only literature written in the English language were considered due to the language barrier.

### 2.2. Inclusion Criteria and Data Extraction

In this review, studies covering the following types of data were included and extracted: the anticancer activity of plant derived bioactive compounds, their structure activity relationship, and the mechanism of their antiviral activities. 

## 3. Plant Derivatives Compounds According to Their Chemical Structure

### 3.1. The Catharanthus Alkaloids

**Figure 1:**
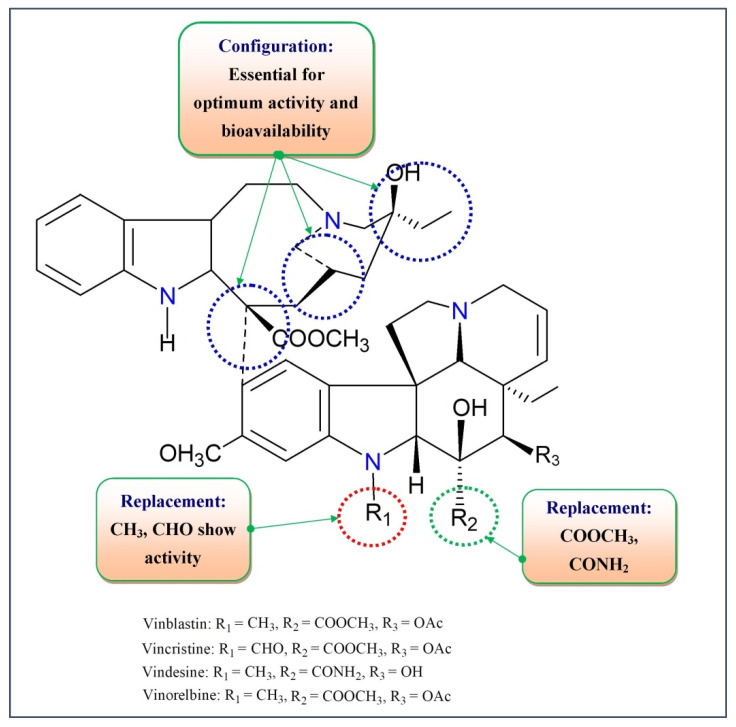
Overview of the SAR and analogs of VAs.

The Catharanthus alkaloids, also known as Vinca alkaloids (CAs or VAs) have covered approximately 130 terpenoid indole alkaloids [10]. Vinblastine (VBL) was the very first alkaloid separated from the periwinkle plant of Madagascar in the 1950s. Vincristine (VCR) and its derivatives are hetero-dimeric (indoloid) alkaloids formed amid the biosynthesis of catharanthine and vindoline and are present in pink *Catharanthus roseus*. Different Catharanthus species which include *C. Trichophyllusand*, *C. Longifolius*, and *C. Lanceus* contain vindoline alkaloids. These alkaloids exhibit a cytostatic impact, abolishing the living cells [11]. This group is comprised of vinblastine, vincristine, anhydro-vinblastine, and the semisynthetic sub-ordinates vindesine (VDS), vinorelbine (VRL), and vinflunine (VFN) (the fluorinated analogue of vinorelbine) (Figure 1). Since 2008, a novel synthetic vinca alkaloid known as vinflunine has been licensed for therapeutic use in Europe [12,13]. Although the structural similarities between the vinca alkaloids are striking, their toxicological profiles are immensely diverse. Although all vinca alkaloids cause peripheral neurotoxicity, vincristine has the highest potential in this instance [14].

Vinblastine and vincristine are now utilized for the treatment of different cancers in the US and other nations, whereas the semisynthetic vindesine is currently in phase II clinical trials for the treatment of hepatocellular cancers, leukemia and non-small cell lung cancer in South Africa [13,14]. Vinorelbine (5’-nor-anhydro vinblastine), a semisynthetic analog, was first developed by Pierre Potier of France and was approved in France in April 1989 and April 1991, respectively, for treating non-small cell lung cancer and breast cancer [15]. The U. S. FDA authorized vinorelbine as a remedy for the non-small-cell lung cancers (NSCLC) in 1995 [16]. Vinorelbine has been proven to have similar effects to vindesine in NSCLC in vivo models. In vivo analysis has shown that vinorelbine iss less effective than vinblastine against breast cancer tumors but as effective as vindesine and vincristine. Based on in vitro and in vivo results, cross-resistance between vinorelbine and other vinca alkaloids is expected to be limited [17,18,19].

**Figure 2:**
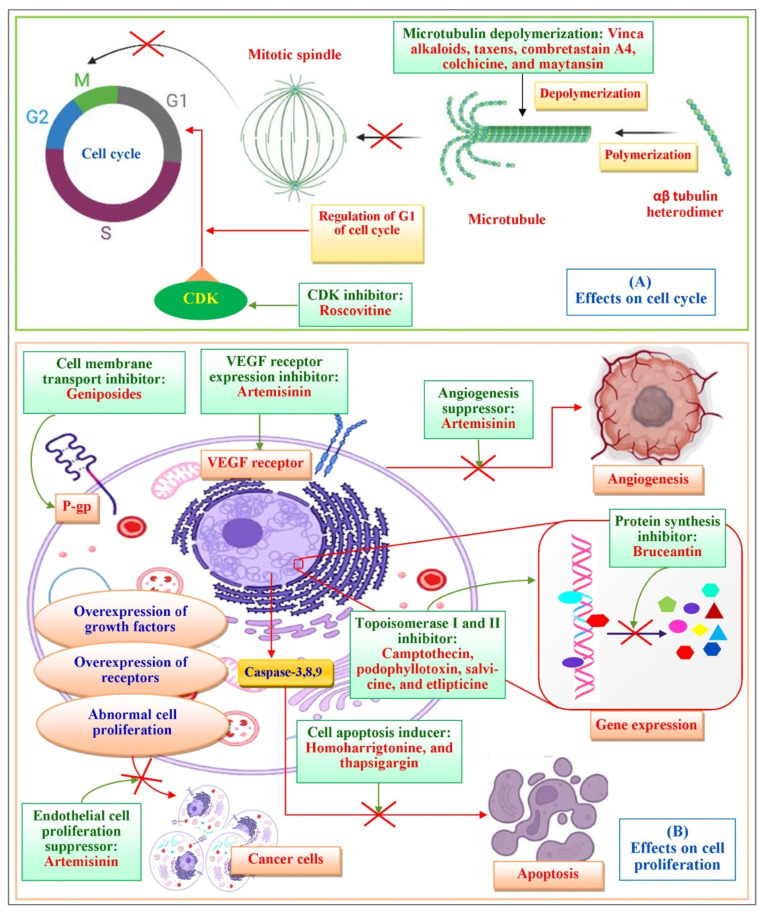
General mechanistic insight of anticancer drugs derived from plants. M, G1, G2, and S represent distinct phases of a cell cycle characteristic to specified functions or intervals; where M phase: mitosis cell division; S phase: DNA synthesis; G1 (gap 1) phase: gap between completion of M phase and initiation of S phase; and G2 (gap 2) phase: gap between completion of S phase and initiation of M phase.

The mechanism of the cytotoxic activity of the *Catharanthus* alkaloids is related to their impact on the microtubules (Figure 2). Their cytoskeletal structures are composed of the heterodimers of α-tubulin and β-tubulin [15,20]. Microtubules shape the mitotic spindle which plays a vital role during mitosis. Hence, microtubules are an appropriate target of multiple anticancer agents, for example, VAs. They target the β-subunit of the heterodimer tubulin [21,22]. *Catharanthus* alkaloids illustrate distinctive activity depending on their concentration. Low concentrations (<1 μmol) suppress the microtubule elements and stabilize them. Although at a greater concentration (>1–2 μmol) they collapse the microtubules and harm the mitotic spindle, they facilitate the inhibition of mitosis and thereby, apoptosis [23]. At lower concentrations, VAs prevent the formation of microtubules through the direct binding at (+) microtubule structure with the resultant impediment in the attachment of GTP and the indirect cross-linking of MAP (microtubule-associated proteins). At high concentrations, they bind to tubules in a unique conformation by forming spirals and *ρ*-crystalline aggregates and cause microtubules to depolymerize. This suppression of dynamics hinders the mitotic spindle from properly forming and decreases tension at the chromosome kinetochores. Mitotic development is slowed because chromosomes are frequently trapped at the spindle poles and are unable to assemble at the equator. The signal from the cell cycle to the anaphase-promoting complex gets inhibited followed by interruption in metaphase to anaphase transition resulting cell death or apoptosis [24,25]. VBL binds at the interface of two tubulin heterodimers (α2–β1) in a head-to-tail rearrangement followed by perturbing polymerization of tubulins leading to crystallization of microtubules as well as mitotic arrest [26]. Vinorelbine was discovered to cause the depolymerization and removal of microtubules in vivo in the P388 murine leukaemia model early in its development [27]. Vinorelbine, like vincristine and vinblastine, inhibited in situ mouse embryo cell growth in the metaphase at lower doses (∼2.0 μmol). This impact was triggered by the gradual depolymerization of interpolar (low concentrations) and kinetochore (high concentrations) microtubules which reduced in quantity but not in length [28,29].

#### 3.1.1. Relevance to Medicine

VAs are frequently utilized as anticancer medications, either alone or in combination with other medicines, to treat a number of cancers, such as breast cancer, osteosarcoma, and acute lymphocytic leukemia. They were first used to treat juvenile hematologic malignancies and then expanded to include solid and adult hematologic malignancies [21,30,31]. Table 1 lists the most important VAs, together with their clinical use and toxicological characteristics. VBL, VCR, and VRL are all on the World Health Organization’s Essential Medicines List [32].

VBL is the best studied of the VAs family and it is used in a variety of chemotherapy regimens to treat Hodgkin’s lymphoma, non-small cell lung cancer, brain cancer, melanoma, and testicular cancer (Table 1) [21,33,34,35]. Although VCR’s therapeutic capabilities have been extensively utilized in clinical application for the treatment of numerous malignancies, its clinical utility is limited by its severe neurotoxicity.

VDS is a semi-synthetic VA derivative that was initially used in clinical oncology. VDS is widely used in chemotherapy protocols, however it has only been approved in a few countries due to its negative effects [36]. VRL is a second-generation VA molecule with a wide range of anti-proliferative action, particularly in advanced breast cancer and advanced/metastatic non-small-cell lung cancer (Table 1).

It was found to be more effective than VBL and VCR while also being less neurotoxic. VRL is accessible in an injectable version, which is frequently utilized in clinical practice. VRL injections, on the other hand, produce venous irritation and phlebitis near the injection site [37]. VFN, a fluorinated VA derivative, is the most recent member of the VA family and is a third-generation molecule. Surprisingly, it is the least neurotoxic of the VAs and shows better anti-cancer action compared to the others [38]. Furthermore, VFN has been licensed in Europe for the treatment of advanced or metastatic transitional cell carcinoma of the urothelial tract [39]. 

In fact, drug-induced perturbation of microtubules may cause an axonal degeneration and a decreased axonal transport, leading to sensory impairments and paraesthesia. Other effects that can occur after a prolonged treatment with VAs include neuropathic pain and loss of deep tendon reflexes, followed by motor dysfunction, ataxia, and paralysis. To overcome drug resistance in anticancer therapy, in the last two decades, VAs have been administered in combination therapies with other drugs acting via a different mechanism of action, rather than being used as single agents. This therapeutic strategy enables a reduction in the VA dosage, thus limiting the toxicological drawbacks, i.e., peripheral neurotoxicity, one of the main side effects related to VAs’ clinical use. Examples of combination therapy include: (a) CVP (cyclophosphamide, vincristine and prednisolone); (b) CHOP (cyclophosphamide, doxorubicin, vincristine and prednisolone); (c) VCRT (vinblastin, cisplatin and radiation therapy); (d) CISCA/VC (cisplatin, doxorubicin, vinblastine and bleomycin); and (e) DOBVD (doxorubicin, bleomycin, vinblastine and dacarbazine) [26].

#### 3.1.2. Viscum Album Extract

Mistletoe is a tiny shrub with leathery leaves that are linear and lanceolar and last for several seasons. In late fall and early winter, its yellowish green blooms turn transparent and produce pale berries. *Viscum album* does not grow on the ground but is transferred to tree trunks by birds. *Viscum album*, unlike other plants, has a 12-month vegetative phase, never touches the ground, and blooms in the winter [40].

Viscotoxins (VT) and lectins collected from the mistletoe plant (Viscum collection), constitute another group of phytocompounds with cytotoxic activity. Viscotoxins are obtained from the extracts isolated from the common mistletoe plant. Viscotoxins are members of the thionin family type III and are characterized by three disulfide bridges [41]. They are cationic proteins, rich in cysteine, and comprising of 46 amino acid residues with six isomers, three of which are viscotoxin A2, A3 and B [42,43].

Viscotoxins can be found inside the leaves and stems of the mistletoe plant. As viscotoxins are hydrophilic, they are present in the aqueous Viscum album L. extracts. The recovery observed with the use of the whole extracts is usually greater than using isolated and purified viscotoxins and mistletoe lectins alone. Viscum album extract (VAE) contains a protein called lectins (mistletoe lectins, ML), ML–1, ML–2, ML–3 D-galactose, oligosaccharides, polysaccharides, N-acetyl-D-galactosamine, and alkaloids [44].

TNF expression is inhibited by the aqueous extract of Korean mistletoe (*Viscum album coloratum*). TNF and other cytokines are produced as a result of an increase in the number and cytotoxic action of natural killer cells. This rise in natural killer cells demonstrates the immune system’s ability to suppress cancer growth without producing negative effects. It is important to remember that TNF has been shown to have a double-edged effect in tumors. For example, higher levels are antitumor, while lower levels cause malignancy, angiogenesis, and metastasis. The antitumor effect of *Viscum album* lectins is attributed to the stimulation of CD4+ T-cell proliferation. The effect of mistletoe triterpene acid dissolved in cyclodextrin on the C.B-17/SCID model of pre-B Acute lymphoblastic leukemia was examined (NALM-6). The findings revealed a dose-dependent activation of apoptosis via caspase-8 and 9-dependent pathways, resulting in a longer mean survival time. Viscum album extracts have anti-cancer properties via regulating the immune system, as well as having direct cytotoxic actions on cancer cells with a weaker effect on normal, healthy cells [45].

They are poisonous to various types of cells. Viscotoxin and mistletoe lectin I show harmful impacts on three tumor cell lines, such as- the T-cellleukaemia cell line Molt4, the Yoshida sarcoma cell line, and the myeloid leukemia cell line K562. Yoshida sarcoma cells are very responsive to the viscotoxin whereas the human leukemia cell line K562 is responsive to the mistletoelectin [46].

Mistletoe treatment is well tolerated. Mild flu-like symptoms and local responses at injection sites are common, dose-dependent, and self-limiting side effects. Allergic responses can occur, and anaphylactic reactions have been reported in a few cases. In human and animal trials, combining VAE with chemotherapy or radiation had no detrimental impact on the rate of remission. Among one study, raw data from a self-selected population revealed a greater incidence of depression in VAE-treated patients, even when baseline imbalances were adjusted. This discrepancy might be attributed to differences in the patient group. For example, the prevalence of harm was significantly different [47].

These drugs or extracts have effectiveness against cancers of the larynx, throat, oral cavity, and breast. Furthermore, for most cancers, the extracts were used for the adjuvant therapy due to their cytotoxic and immunostimulatory properties [48]. These results are more obvious with the use of complete extracts, rather than the use of the isolated viscotoxins and mistletoe lectins alone. Viscotoxins are further able to act as an immuno-modulatory on granulocytes and to improve cytotoxicity mediated by the natural killer cells [49,50].

### 3.2. Taxanes

**Figure 3:**
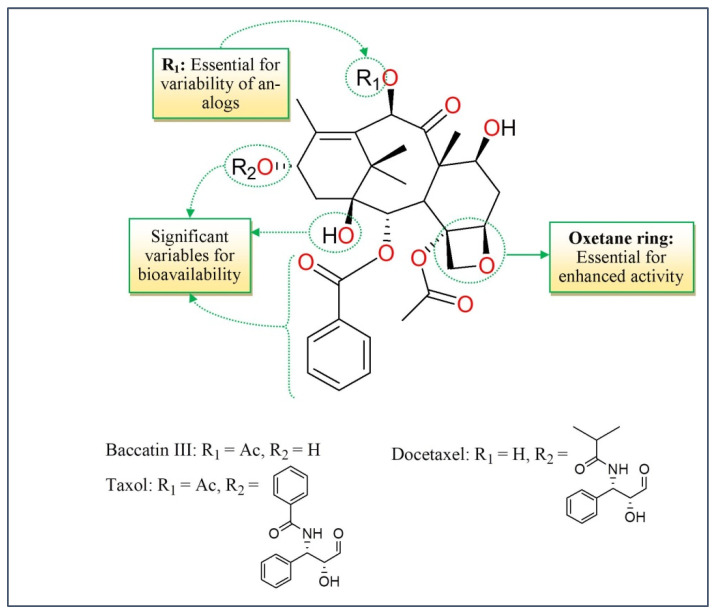
SAR and analogs of taxanes.

Taxol, a particularly promising chemotherapeutic diterpene effective against ovarian and breast cancers, was separated from the bark of European yew (*Taxus baccata*) and/or the Pacific yew (*Taxus brevifolia*) tree needles. It has a place amongst a plethora of compounds known as teroenes which are inhibitors of mitosis [51]. Taxol has a 6-8-6 tri-ring taxane skeleton with nine chiral centers (Figure 3) whereas the semisynthetic taxanes have found to be derived from ten rings compounds [52,53].

The anticancer movement of taxanes is comparable to the activity of vinca alkaloids and is related to their impact on the microtubules, which are composed of heterodimers of α-tubulin and β-tubulin. Taxanes constitute the microtubule-interacting and microtubule-stabilizing agents that potentiate the microtubule polymerization [12] (Figure 2). The approach of microtubule polymerization and depolymerization are fundamental for mitotic cell division. Taxanes, rather than vinca alkaloids, bind to the inside of the microtubules with high affinity. An excessive concentration of taxanes causes microtubule polymerization, whereas the Catharanthus alkaloids repress it. Taxanes are one of the most effective drug types utilized for the remedy of breast and ovarian cancers and are also utilized to treat squamous cell carcinoma of the neck and head [54].

### 3.3. Camptothecin

Camptothecin and its subordinates have a place in the pentacyclic quinoline alkaloids. Camptothecin (CPT) and 10- Hydroxycamptothecin (OPT) were initially obtained from the wood, bark, and fruits of the Chinese *Camptotheca acuminata* (Nyssaceae) tree and have anticancer potential [55]. The lactone ring of camptothecin is relatively sensitive to hydrolysis that results in the generation of carboxylic acid derivatives. Hydrolysis can lessen the antitumor activity of camptothecin [56].

Another drawback of camptothecin is its poor hydrophilicity; although its water-soluble semisynthetic subordinates, topotecan and irinotecan, have clinical applicability. The issue of hydrolysis within the camptothecin subsidiaries was overcome with the addition of a methylene group into the α-hydroxylactone ring, thereby generating a seven-membered stable ring within the compound (e.g., in diflomotecan). 

**Figure 4:**
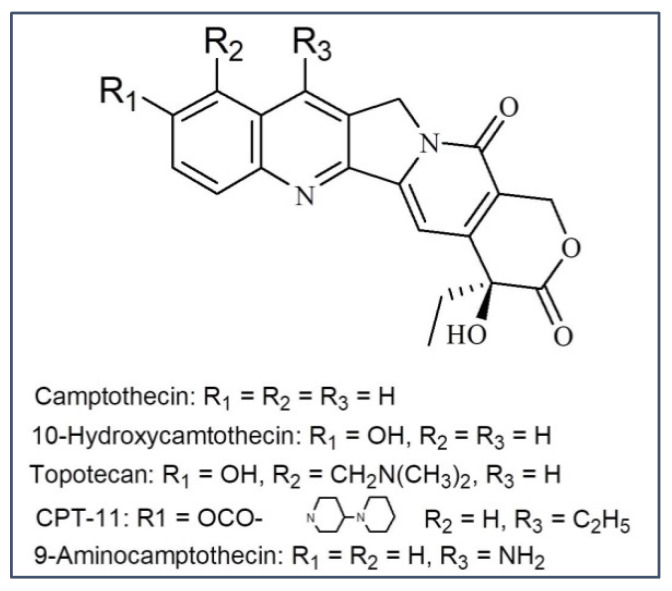
Analogs of camptothecin.

SAR study of CPT analogues established a point-to-point association between CTP analogue’s potency to hinder topoisomerase I catalytic effectiveness and their cytotoxicity [57,58,59,60]. The improvement and utilization of the derivatives of camptothecin as an anticancer drug regularly suffers from their insufficient water solubility. As of late, a hydrophilic 7-(aminoacylhydrazono) previously known as camptothecins, have been synthesized and assessed for their capacity to cause protein-related DNA breaks and hinder topoisomerase I action [61]. While comparing with camptothecin, five of the compounds were as powerful or stronger in these assays but were less poisonous in a few cancer cells lines, thereby proposing that the 7-position within the B ring may be a reasonable area for incorporating a polar moiety into camptothecin and thus, generating analogs with upgraded topoisomerase I inhibitory potential. To preserve the function of pyridine, the 5-position of the C ring ought to be free of any steric hindrance from both the ɑ and β sides; hence, substituents at this position may decrease activity [62] (Figure 4). 

The cytotoxic potential of this group results from the reversible breakage of DNA strands during the natural cellular cycle. Camptothecin is associated with a complex of topoisomerase I and DNA, thereby hindering the reassembly of a single chain DNA strand [63,64]. At this point, camptothecin intercalates among the nitrogenous bases inside the DNA strands. The combination of the camptothecin medicate with the DNA–topoisomerase complex inhibits the formation of the bonds at the sites of nicks and harms the structure of the double stranded DNA chains. The double-strand breaks introduced through the replication may be the primary motive for the DNA damage inside the tumor cells treated with camptothecin and its derivatives, which are the drugs that particularly act on the S stage of the cell cycle [65] (Figure 2).

### 3.4. Combretastatin

**Figure 5:**
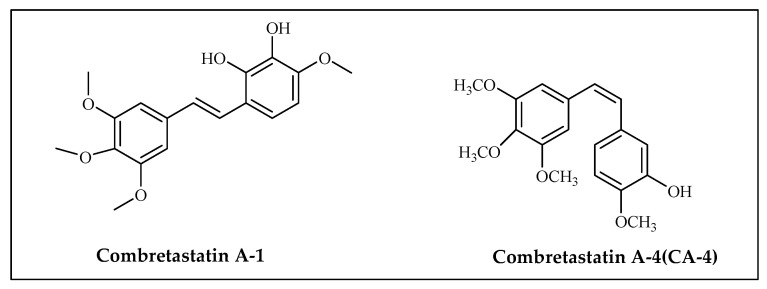
Combretastatins with anticancer activity.

Combretastatin could be a stilbene subordinate that is displayed within the *Combretum caffrum* tree, additionally referred to as the “African willow”, which grows inside the Southeastern area of Africa. As a stilbene by-product, it could exist in trans (e.g., combretastatin A–1 (CA–1)) and cis (e.g., combretastatin A–four (CA–4)) bureaucracy Combretastatin A-1 (Figure 5). Combretastatin A-4 and its derivatives have been synthesized by means of Pettit and associates in 1987 [47]. Currently, the synthesis of the latest subordinates of podophyllotoxins has been carried out and their activities were assayed on skin carcinoma A431, human cervix adenocarcinoma HeLa, breast cancer cell lines (MCF7, MDA-MB-231), lung carcinoma A549, and ovarian cancer cells SKOV. These compounds productively repress tubulin polymerization with IC_50_ values underneath 1μM [66].

Current SAR studies have demonstrated some basic highlights of bioactive CA-4 subordinates. For example, the cis-olefinic bridge among ring A and ring B is essential for activity whereas the 3,4,5-trimethoxy bunch on ring A and 4-methoxy group on ring B are favored for solid movement. In expansion, 3-hydroxy substitution on ring B was suggested to retain activity. Present SAR study stated that substitutions with bromine or fluorine atom can also be possible without a significant lack of activity [67,68,69,70]. 

Combretastatins are microtubule-targeting agents, the same as taxanes and vinca alkaloids. These compounds are also known as vascular disturbing agents (VDAs) which hinder the angiogenesis of the tumor blood vessels. The component of activity of combretastatin as an antineoplastic compound is related with its authoritative to β-tubulin at what is referred to as the colchicine site, inflicting the destabilization of the microtubules and mitotic arrest. Even as the combretastatin A–4 isomer is stronger than a tubulin folio, the combretastatin A–1 trans isomer exhibits a more grounded cytotoxic impact [71].

### 3.5. Podophyllotoxin

**Figure 6:**
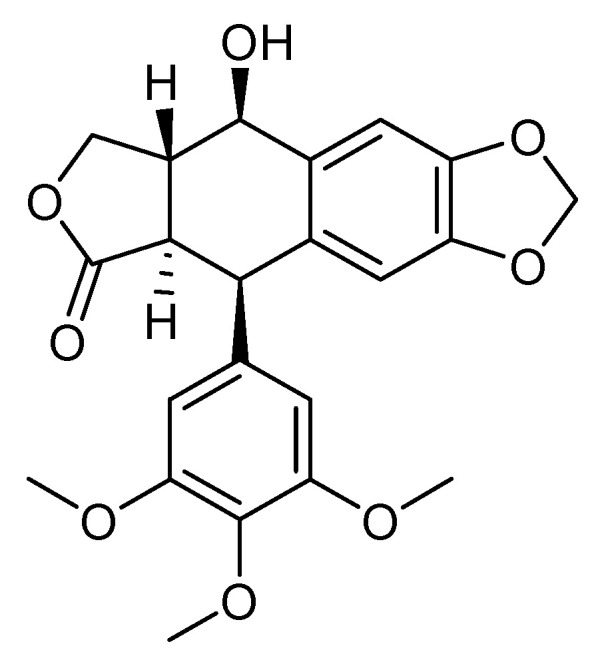
Podophyllotoxin.

Podophyllotoxin (Figure 6) is a toxin lignan separated from the Berberidaceae family (i.e., Podophyllum). The gum, which is called podophyllin, is derived from the *Podophyllum peltatum* species found in North America. Podophyllotoxin is derived from *Podophyllum emodi* gum which is commonly found in Asia [72]. *Podophyllum peltatum* plant life also incorporates ɑ-peltatin, β-peltatin and their corresponding glycosides in conjunction with podophyllotoxin- ɑ -D-glucoside. Podophyllotoxin and its derivatives boast substantial effects as organic antineoplastic drugs and antiviral agents. Podophyllotoxin and its glycosides display a solid cytostatic impact because they disturb the organization of the karyokinetic shaft.

These compounds connect to the colchicine space of tubulin and stop tubulin polymerization. In expansion, the podophyllotoxins interacts with DNA topoisomerase II leading to break down of DNA strands resulting cell-cycle arrest at G2-phase. This action is interceded via the arrangement of a steady complex with DNA and topoisomerase II. Etoposide and teniposide are the semisynthetic subordinates of podophyllotoxin and show cytostatic properties by inhibiting topoisomerase II [73]. Etoposide has observed to work against brain tumor in combination therapy with cyclophosphamide, cisplatin, vincristine, and carboplatin [74,75]. Etoposide and teniposide settle the halfway covalent complexes shaped between topoisomerase II and the break in DNA, as well as the acceptance of DNA damage which captures the cell cycle in metaphase and leads to apoptotic forms. Podophyllotoxin is additionally utilized for the semisynthesis of anticancer drugs, such as azatoxin, etoposide, etopophos, and tenioposide, as well as GL331, NK611, tafluposide, Pand TOP-53 [76,77,78]. Podophyllotoxin subsidiaries are utilized within the treatment of various cancers, such as lymphomas, sarcomas, neuroblastomas, testicular and ovarian cancers, gastrointestinal cancer, brain tumors, nasopharyngeal carcinomas, cervical carcinomas, osteosarcomas, colon cancer, prostate cancer, breast cancer, testicular carcinomas, and small-cell lung cancer [79]. Due to toxicity of podophyllotoxin, it is limitedly used in anticancer therapy. In contrast, its subsidiaries, along with etoposide and teniposide, are utilized in treatment for testicular cancer, lung cancer, gliomas, and lymphomas [73]. Podophyllotoxin subordinates are also utilized within combination treatments; for example, in combination with cisplatin within the first-line chemotherapy for extensive-stage SCLC or small-cell lung cancer [80].

### 3.6. Geniposide and Their Derivatives

**Figure 7:**
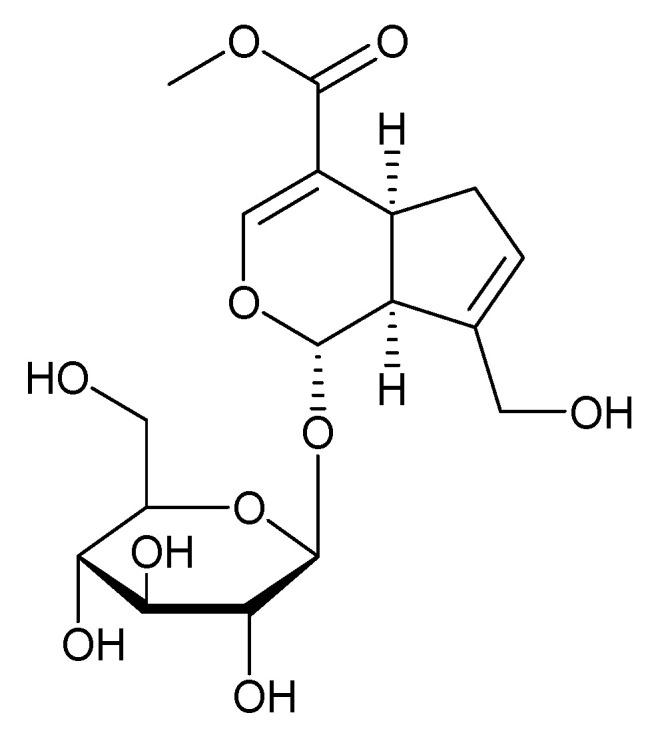
Geniposide.

With an atomic formulation of C_17_H_24_O_10_, geniposide is a small-molecular-weight (388.366 Da) compound (Figure 7). Geniposide (GS) is found in about 40 species of plants. However, the foremost source of geniposide is *Gardenia jasminoides* Ellis (Rubiaceae) which have been utilized in Chinese pharmaceutical for centuries. Various species of this genus and alternative members of the Rubiaceae family are known to contain geniposide. At present, approximately 90 distinctive subordinates of the geniposide backbone are known. Genipin is an aglycone that is formed by means of the hydrolysis of geniposide which takes place within the identical plant fabric in conjunction with its other derivatives, such as geniposidic acid [81]. 

The level of sugar (glucose) moiety within the particle gives the compound its extremity with great water dissolvability and subsequently, superior anticipated bioavailability compared to its aglycone (genipin). The partition coefficient (P) of geniposide on the premise of the octanol/water system is detailed as 0.1077, whereas its log p value is 0.97. Thus, the compound may not be promptly passing via cell membranes [82]. 

When administered orally or intraduodenally, geniposide had good choleretic effects. When injected intraportally, however, this effect was prevented. Genipin, the aglycone of geniposide, on the other hand, had favorable choleretic effects when administered intraportally or intraduodenally. Furthermore, in vitro and in vivo investigations have shown that the intestinal microflora enzymes (-glucosidase) in the intestine may convert geniposide to genipin. This shows that geniposide has no cholagogue action until it is converted to genipin, another active metabolite. In the presence of ammonia, genipin can be converted to genipinine, a novel nitrogen-containing molecule. Despite the fact that both geniposide and genipin contain methyl ester, which seems to be hydrolyzed to genoposidic acid, bacterial and animal esterase could only hydrolyze genipin to the aglycone of genoposidic acid. Geniposide was not stable in duodenum perfusions but was stable in jejunum, ileum, and colon perfusions, which might be due to differences in intestinal contents. Geniposide, on the other hand, was shown to remain stable in different areas of the gut once the pH values of perfusions were adjusted to 7.0. These two occurrences suggest that acidic circumstances may impact geniposide indirectly by influencing intracellular contents, but not directly by reducing geniposide stability. Adding verapamil, a well-known inhibitor of P-glycoprotein (P-gp), increased geniposide absorption by 2.4-fold, while EDTA, a common inhibitor of cell membrane transport channels, had no effect, suggesting that geniposide transport is linked to P-gp rather than membrane transport channels. Intriguingly, another traditional medicine, notoginsenoside R1, was able to dramatically increase the intestinal absorption of geniposide (1.424 mg/mL) by 1.7- and 1.4-fold in a manner comparable to that of a P-gp inhibitor at doses of 0.1 and 0.2 mg/mL [83].

Genipin and geniposide have proved to show an antidiabetic impact, as well as neuroprotective results in neurodegenerative infections (e.g., Alzheimer’s disorder) [84]. The subsidiaries of iridoid glycoside geniposide are a generally novel group of compounds with antitumor as well as anticancer activity. The work additionally mentioned the antioxidant and pro-oxidant mechanisms of these compounds, the technology of cell-cycle regulation, reactive oxygen species, and the pro-inflammatory activity of the compounds [85]. It has been mentioned that genipin shows a solid apoptotic cellular dying effect impact in human non-small-cell lung cancer H1299 cells [86]. *Gardenia jasminoides* (Cape jasmine) is widely used in Chinese traditional medicine for the treatment of inflammation, brain disorders and hepatic disease [87]. A large amount of research in animal and cell models has indicated the potential of geniposide for the treatment of a wide range of illnesses, ranging from neurological diseases to cardiovascular diseases and diabetes mellitus (DM). However, in terms of the therapeutic use of geniposide, the following issues must be addressed immediately. For example, investigations on geniposide are currently limited to animal and cell levels and have not been conducted in the human body. As a result, more clinical research is needed to determine the definitive function of geniposide in human therapies. Furthermore, despite significant advances in the pharmacokinetics of geniposide, additional information on its transformation, monitoring, and metabolism is required to ensure its efficacy and safety. Researchers examined the hydrolysis of geniposide to genipin in an aqueous-organic two-phase system using -glucosidase immobilized via crosslinking-embedding. Immobilized and free -glucosidase had optimal reaction pH values of 4.5 and reaction temperatures of 55 °C and 50 °C, respectively. Immobilization also reduced the influence of pH variations on -glucosidase activity and improved its heat resistance. Second, natural medications frequently contain low concentrations of active substances, which are difficult to detect after entering the peripheral circulation. As a result, we should use a range of approaches and integrate different indications to improve the monitoring program. Third, there is currently little research focusing on the metabolites of geniposide. As a result, we should investigate the structure of its metabolites, metabolic pathways, and pharmacological effects in order to uncover the actual molecular basis of geniposide’s pharmacological activities. Fourth, in order to obtain high geniposide content at little cost, additional plants and their components must be identified in order to identify the best candidate for geniposide preparation. All of the aforementioned factors will be critical in the development of novel drugs and their clinical use [88]. As a bioactive natural product, GS has the potential to be developed as a candidate medication or lead chemical. However, a significant amount of research is still necessary. We have reviewed recent research on GS in different study disciplines, including phytochemistry, pharmacology, pharmacokinetics, and toxicity, in this review. Despite these important discoveries, several issues remain unsolved, which have been raised here and require immediate attention. This iridoid glycoside will be used in a variety of ways, whether for basic research or application studies [81].

### 3.7. Colchicine

**Figure 8:**
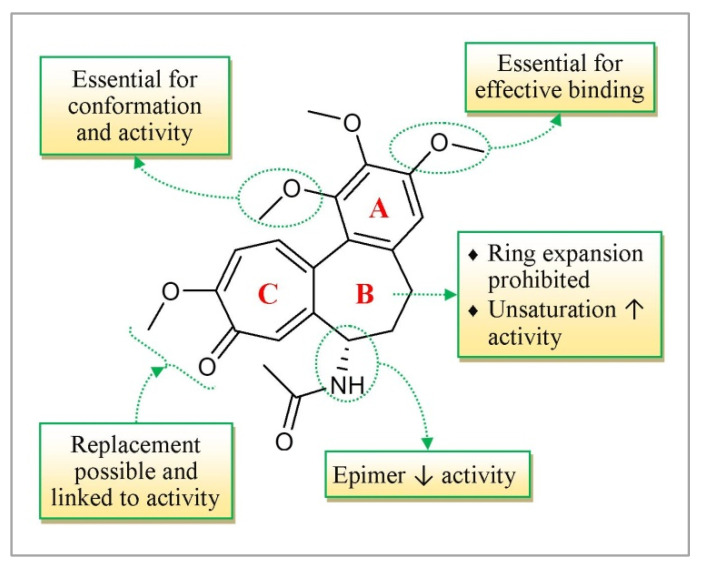
SAR of colchicine; ↑ and ↓ indicate increase and decrease respectively.

Colchicine is an alkaloid isolated from the plants of the Colchicum genus (e.g., the harvest time crocus, *Colchicum autumnale*, also known as “meadow saffron”) and utilized in the treatment of gout and familial Mediterranean fever (FMF) [89,90]. Inquiry has uncovered that colchicine and its analogs also appear to possess anti-inflammatory, antitumor, and anti-HIV activities [91,92]. The SAR of colchicine is presented in Figure 8.

Comparative to vinca alkaloids, the component of activity of colchicine is based on the depolymerization of the microtubules at excessive concentrations and the adjustment of the microtubule elements at poor concentrations. Most of the antimitotic drugs (such as- podophyllotoxin and combretastatin) tie to the colchicine space of tubulin and destabilize then within the lowest-viable effective concentrations. Colchicine may also regulate the voltage-dependent anion channels of the mitochondrial membrane and improve the cellular-free tubulin to restrain the mitochondrial digestion system within the cancer cells [93]. The dynamics of mitotic spindle microtubules are also stabilized by colchicine. Microtubule dynamics stabilization is linked to cell cycle arrest during mitosis in sensitive tumor cells which leads to cell death via apoptosis. 

Colchicine, as a mitotic inhibitor, is more effective against cancer cells than normal cells because cancer cells are more vulnerable to poisoning [89] (Figure 2). Detachment of the cancer cell from the primary tumor through changes in cell–cell and cell–ECM adhesion, degradation of the ECM and basement membrane, invasion of tumor cells into peripheral tissue, and finally, intravasation into the blood or lymphatic vessels and attachment at the target tissue are all steps in tumor cell metastasis [94]. Cell adhesion, migration, and invasion are the primary contributions to metastatic dissemination in these critical stages. Colchicine inhibits cell migration, adhesion, and invasion in hypopharyngeal cancer cells, resulting in substantial anticancer effects [95]. The anti-proliferative impact of colchicine on hepatocellular carcinoma cells is roughly 200-fold less than that of epirubicin. A small amount of colchicine has been verified as clinically suitable within the palliative treatment of hepatocellular carcinoma [89] and cholangiocarcinoma [94]. Excessive toxicity has constrained the clinical utilization of colchicine. Nonetheless, colchicinamide, a semisynthetic derivate of colchicine in which the C-14 methoxy bunch is supplanted via an amino bunch, confirmed a healing index 1.75- crease more prominent than that of colchicine against different strong tumors. Colchicinamide has been utilized in China for the treatment of breast cancer and other strong tumors [95]. Deacetylcolchicine, which appears to show viability against Hodgkin’s lymphoma, melanoma, and chronic granulocytic leukemia, is presently in stage II clinical trials [96]. As of late, an arrangement of novel deacetamidothiocolchicine-7-ols and their esters have been established as antitubulin agents. These compounds provided powerful inhibitory action against tubulin polymerization and specific cytotoxicity against colon and CNS cancers, in addition to melanoma in vitro [97].

### 3.8. Artesunate

**Figure 9:**
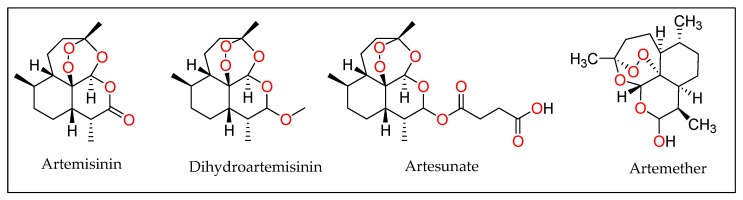
Artesunates with anticancer activity.

Artesunate may be a semi-synthetic subordinate of artemisinin collected from a Chinese plant *Artemisia annua* L. therapeutic (Asteraceae) [98]. It is believed to have first been described by the Chinese during the Jin dynasty around 317–420 AD due to its medicinal properties, specifically, for reducing fever. This clinical scenario would later be identified as malaria, an infectious disease caused by the Plasmodium parasite. Although records show that malaria was treated for more than two thousand years with this compound, it was not until 1969 that alternative treatments were sought out because resistance developed against chloroquine and quinolones. Dr. Youyou Tu was commissioned by the Chinese government to find an alternative cure for Malaria. By 1972, clinical trials with artemisinin were held for the first time on human patients. Thirty patients that were infected with malaria were cured with the extract, showing no signs of fever or parasites in the blood. Dr. Youyou Tu et al. isolated and discovered artemisinin and its chemical structure in the same year [99]. It was utilized as an antimalarial medicine. It has been proven that artesunate shows anti-angiogenic activity against certain forms of cancer. Dihydroartemisinin (DHA) is a reduced product of artemisinin and it is the first metabolite of artemisinin and its subordinates. DHA is water dissolvable, generally unsteady and vulnerable to oxidation reactions due to the lactol moiety in its structure. Artesunate is a form of hemisuccinate ester of dihydroartemisinin (Figure 9). It is miles polar and has bad stability in aqueous solution because the effortless hydrolysis of its ester linkage occurs quickly, which cases the drug to go through oxidative reactions with ease. Artmether is a form of methyl ether of artemisinin. The methyl substituent in artemether makes the compound chemically solid and more lipophilic. Hence, DHA inflicts maximum results and artmether generates the least effect. Rutterman and colleagues conducted a trial with 23 dogs with surgically unresectable tumors to assess both antineoplastic efficacy and its potential side effects. The study also looked at the in vivo and in vitro responses of cancer cells to artesunate using four cancer cell lines. Artesunate or dihydroartemisinin were given to primary cancer cell line cultures. All four neoplastic cell lines were sensitive to the therapy, with one cell line being more resistant to dihydroartemisinin treatment. The 23 dogs were given artesunate at a dose of 651–1178 mg/m2 for 7–385 days in the same trial. There was no cardiotoxicity or neurotoxicity in any of them. Seven dogs showed no signs of toxicity or side effects, while 16 dogs experienced fever, gastrointestinal toxicity, or hematologic toxicity. Artemisinin and its subordinates stop the development and expansion of cells and specifically kill tumor cells. As of late, numerous unused artemisinin subsidiaries have been depicted, and it is conceivable that many of them will be compelling against cancer. It also has confirmed activity against persistent leukaemia. These compounds have proved to be less harmful and are promising specialists in antileukemia chemotherapy [99,100]. 

In vitro and in vivo, antimalarial ART suppressed angiogenesis by reducing endothelial cell proliferation and differentiation or lowering VEGF receptor expression (Figure 2). Because of their anti-angiogenic properties as well as their low and selective toxicity, artemisinin derivatives might be potential new options for angiogenesis suppression. It has been suggested that artesunate has potential ROS generating activity against chemoresistant neuroblastoma cells. The cytotoxicity activity of artemisinin derivatives has been linked to the production of reactive oxygen species and carbon-centered ART free radicals, both of which have an impact on cellular health parasite proteins and lipids as well as with the baseline mRNA expression of genes from several gene families such as- cell cycle regulator genes, growth regulator genes, oncogenes, tumor suppressor genes, and so others. However, artemisinin’s mechanism remains unknown. Apoptosis caused by derivatives is poorly understood. Further research is required [99,101].

### 3.9. Homoharrigtonine

**Figure 10:**
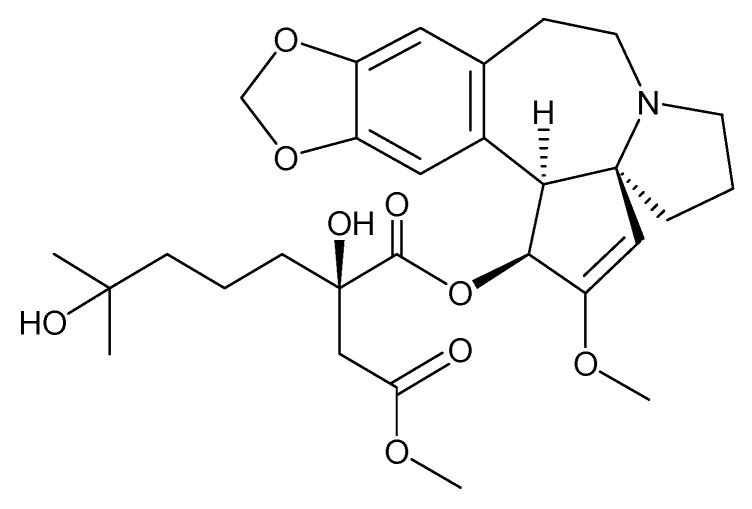
Homoharringtonine.

Cephalotaxus alkaloids consist in harringtonine, isoharringtonine and homoharrigtonine (Figure 10) which are found from few Cephalotaxus (Cephalotaxaceae), including *C. harringtonia* K. Koch, and *C. haianensis* qinensis, all of which are utilized in conventional Chinese medication. The first synthesis of homorringtonine was carried out via Hiranuma and Chudlicky (1982) [102].

The activity of these drugs is associated with the square of amalgamation within the peptidyl transferase centre and ends in cell apoptosis [103]. Homoharringtonine has shown promise in various haematological cancersand it was widely investigated in the 1980s, displaying an intriguing effectiveness in CML as well as AML When homoharringtonine was administered, however, hypotension was the DLT at large dosages and/or infusions on a tight schedule, whereas DLT for continuous low-dose infusions was myelosuppression. Nonhaematologic toxicity was thought to be minor and reversible [104].

Harringtonine and homoharringtonine limit protein translation by interfering with the ribosomal A-site which prevents the first elongation phase of protein synthesis (Figure 2) [105,106]. The fast loss of proteins in short time periods is a significant short-term impact of homoharringtonine on cells’ half-lives [107]. Several proteins are involved in cell survival. The genes for proliferation and proliferation with short half-lives are encoded by G/C-rich 5′ UTRs on mRNAs and have complicated three-dimensional structures [81]. Homoharringtonine has been clinically trialed in progressed breast cancer, intense myelogenous leukemia patients, and in patients with myelodysplastic disorder (MDS) and MDS advancing intense myeloid leukemia [108].

### 3.10. Salvicine

**Figure 11:**
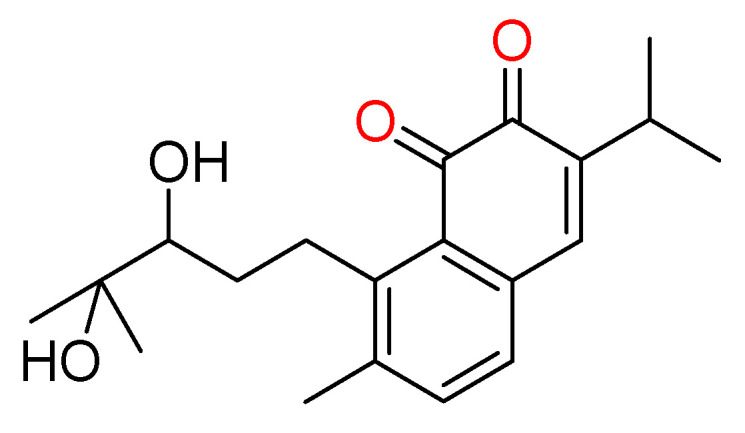
Salvicine.

Salvicine (Figure 11) may be an altered diterpenoquinone subsidiary confined from the Chinese herb *Salvia pronitis* Hance (Labiatae). This compound was chemically synthesized by Sheng et al. in 1999 and it has exhibited strong inhibitory action on a wide array of human tumor cells in vitro and in mice bearing human tumor xenografts [109]. Salvicine and its subordinates are non-intercalative topoisomerase II poisons that work against solid tumor [110].

Salvicine initiates the breakage of the double strands of DNA by encouraging TOP2 movement, hindering the re-ligation related with the restraint of tumor development. In human most cancers cells, salvicine induces damage to precise DNA genes, driving apoptosis (Figure 2). Moreover, reactive oxygen species have proven to play a crucial role within the salvicine-triggered cell response, inclusive of TOP2 inhibition, DNA damage, dodging MDR and tumor cell grip restraint [111].

In contrast, there was no inhibitory activity against TopoI’s catalytic activity. Salvicine is as cytotoxic as VP-16 but weaker than VCR in three leukemia cell lines after 72 h of treatment, according to microculture tetrazolium (MTT) tests. Salvicine is over 5.41- and 4.15-fold more effective against 12 lines of solid tumor cells than VCR and VP-16 [110,112].

Particularly, Salvicine presents better effects against gastric and lung carcinoma cells. Moreover, the antitumor effect of salvicine was found to be associated with its ability to induce apoptosis in K-562 and SGC-7901 cells [111]. Salvicine’s anticancer efficacy was also tested in animal models. Salvicine has anti-cancer efficacy in mice with S-180 sarcoma and Lewis lung cancer, as well as human lung adenocarcinoma xenografts A-549 and LAX-83 [109].

### 3.11. Ellipticine

**Figure 12:**
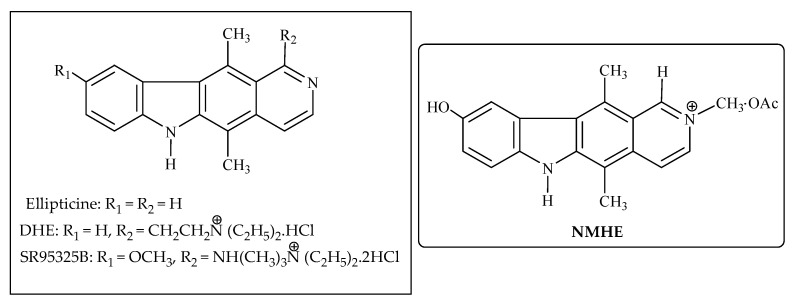
Ellipticine and its analogs.

Ellipticine (5,11-dimethyl-6H-pyrido-(4,3-b)-carbazole) is an alkaloid (Figure 12) that shows antineoplastic activity, and is separated from *Ochrosia elliptica* leaves (family: Apocyanaceae) as well as from a few other species of Ochrosia, together with *O. acuminata* and *Bleekeria vitiensis*. Synthetic ellipticine, along with some soluble byproducts, shows impressive antitumor action [23,113,114].

Ellipticine acts by binding to DNA, shaping covalent bonds, and/or to topoisomerase II and finally, leading to hampering the cell cycle by directing the expression of a few kinases, such as cyclin B1 and Cdc2 (Figure 2). Additionally, they can trigger the phosphorylation of cyclin, including apoptosis and the generation of cytotoxic free radicals. Ellipticine also has an inhibitory or stimulatory effect on biotransformation enzyme alongside its genotoxic and pharmacological impacts through modulation. Ellipticine shows cytotoxicity against human breast cancer cells (MCF-7), leukemia (HL-60 and CCRF-CEM) cells, neuroblastoma (IMR-32, UKF-NB-3 and UKF-NB-4) cells, and glioblastoma cells (U87MG). The pharmacological effectiveness of this compound depends on its metabolism by cytochrome P450 (CYP)- and/or peroxidase-mediated metabolism with covalent DNA adducts [115,116,117]. Three congeners of this compound have been clinically investigated. One of these congeners, (N-2-(diethyl amino-ethyl)-9-hydroxy ellipticinium chloride) or DHE, acts by intercalation. It was investigated in stage II clinical trial in Europe for the treatment of breast cancer by administering in weekly regimen [118,119].

A phase II trial of NMHE (2-N-methyl-9-hydroxy-ellipticine) given as 100 mg/m^2^ week by week was performed in 57 patients with progressed metastatic breast cancer [120]. SR95325 B (Retelliptine dihydrochloride, NSC D-626717-W) is another ellipticine derivative with a significantly high level of antitumor action in resistant murine strong tumor models. It was included in a phase I trial [121].

### 3.12. Roscovitine

**Figure 13:**
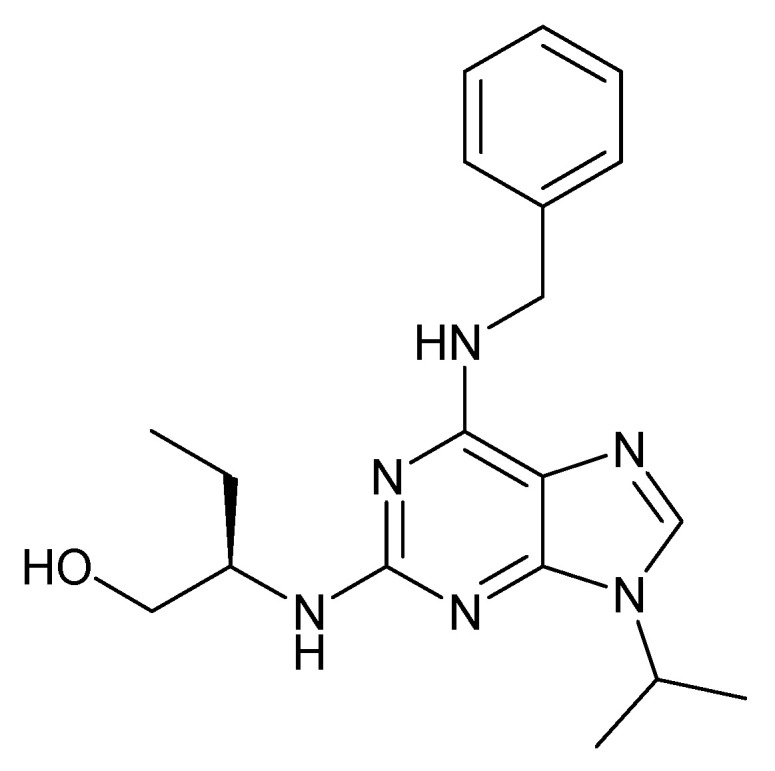
R-roscovitine.

Another anticancer compound could be a purine-based, semisynthetic congener of R-roscovitine (Figure 13), which is isolated and collected from the cotyledons of *Raphanus sativus* L. (Brassicaceae). This promising member of cyclin-dependent kinase (CDK) inhibitor was synthesized by Meijer et al. (1997) [122]. Previous reports showed that roscovitine and its three available structural analogue acts as potent N-type calcium channel agonists. A four-quarter method was used to evaluate the literature outcomes and to guide the preliminary steps of SAR studies. Particularly, zone one was superficially probed in the inert analogues which varied from the purine scaffold of roscovitine. However, shifting of the nitrogen in pyrazolo(1,5-a)-1,3,5-triazine 3 has retrieved the agonistic activity on N-type Ca2+ channel. Very little information can be found on the analogues of zone two, e.g., (R)-olomoucine II. The previously trisubstituted analogues were inactive and suggest the importance of hydrogen atomat N-6. The presence of phenol at 6 substituted the presence of a benzene ring on roscovitine with a negligible loss of effectiveness. Hence, it can be concluded that zone two is open for further amendment. On the contrary, bohemine and (S)-roscovitine demonstrates the necessity of zone three for the anticipated N-type Ca2+ channel agonist activity. However, minor modification in zones three in nine, resulted in a notable drop in activity [123,124,125].

Roscovitine, a powerful CDK/cyclin E inhibitor, has proved to exhibit broad spectrum anticancer activities. Roscovitine was studied under phase II clinical trial on patients with lung and breast cancers. Seliciclib has been shown to suppress RNA-polymerase-II dependent transcription and down-regulation of the protein MCL1 [126]. Olomoucine is a cyclin-dependent kinase inhibitor (CDK4). In turn, roscovitine precisely inhibits CDK5 activity with simultaneous inhibition of MDA-MB231 cell proliferation and induction of apoptosis. In contrast, olomoucine does not distress MDA-MB231 cell proliferation and apoptosis; however, roscovitine-mediated reticence of proliferation is irrevocable. The probable mode of action of roscovicine and its congeners is related to the inhibition of cyclin-dependent kinase activity which favorably constrains numerous target enzymes, such as CDK1, CDK2 and CDK5, leading to cell-cycle halt in the G1 and G2 phases [127,128,129,130] (Figure 2).

### 3.13. Maytansin

Maytansin or maytansine was isolated in the 1970s from *Maytenus serrata* (Celastraceae) or Maytenus ovatus and it was synthesized by Corey and co-workers (1980), Itacts as a cytotoxic substance [131]. Maytansin and its derivatives are from the maytansinoids group of naturally occurring antimitotic compounds, which act by inhibiting microtubule assembly close to the vinblastine binding region [132]. 

Maytanasin exerts anticancer activity by similar mechanism as vinca alkaloids (Figure 2). Maytansin has a history of being used as an effective treatment (in vivo) against Lewis lung carcinoma and B16 murine melanocarcinoma solid tumors and has antileukemic action against P388 murine lymphocytic leukaemia. The maytansinoids inhibit the polymerization of tubulin by forming bonds with the vinca domain of tubulin next to the vinca site. Thus, the binding of maytansine at this region suppresses the vinblastine binding and stops the association of GTP with tubulin. This binding site is shared by rhizoxin [133,134,135]. In addition to several naturally occurring maytansinoids, a number of semisynthetic equivalents have been prepared to establish an SAR [136,137]. Even if the presence of a C-3 ester is vital for activity, fundamental variability of the side chain is allowed. Furthermore, the presence of the C-9 carbinolamide is crucial for antiproliferative activity. Multiplying cells are more sensitive to maytansine than the nonproliferating ones.

**Figure 14:**
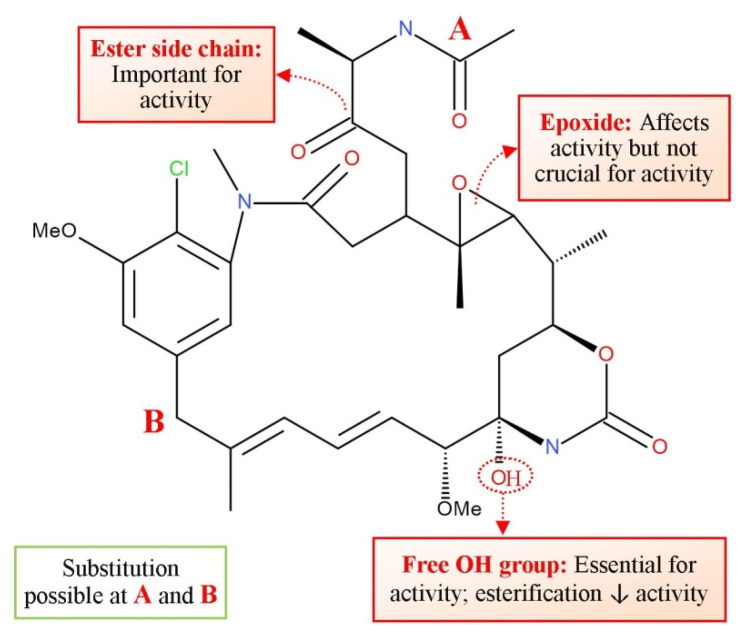
SAR of maytansine.

Suitably designed maytansinoid agents can have potent cytotoxic activity, and at the same time, virtuous aqueous stability and reasonable solubility. Structure–activity relationship studies of maytansinoids indicated that the requisite of the C3 ester side chain is for biological activity but the side chain can be modified without a significant loss of activity (Figure 14). The insertion of methyl disulfide substituent into the C3 ester side chain has resulted in maytansinoid derivatives which show either similar or enhanced activity. Reduction of the methyldisulfide led to the formation of a thiol group that enabled linkage to antibodies. Although maytansin acts on the cancer cells, its clinical utilization has been troubled by severe unwanted effects and negligible efficiency. However, its congeners, especially maytansin analogues linked to antibodies (maytanosoids), are used in various types of cancer chemotherapy [138].

Nevertheless, maytansinoids were publicized to be 20 to 100 times more potent than vincristine in vitro. The mode of action of maytansinoids is attributed to their high attraction to receptors articulated on the exterior of cancer cells. The HuN901 monoclonal antibody conjugate with maytansin showed high affinity for CD56 [139]. 

Initial research revealed an elevated mitotic index and DNA content typical of the Gz t M stages of the cell cycle. Furthermore, maytansine entirely prevented the cleavage of sea urchin eggs at low doses. The impact of the macrolide on vincristine-binding to tubulin was studied since there was cross resistance between vincristine and maytansine in some cell lines. Remillard et al. also discovered that microtubules depolymerize when they interact with a high affinity location [140]. However, whereas binding studies may easily detect interaction at low affinity sites, aggregation does not occur, and vinblastine’s aggregating function is prevented. Maytansine, unlike vinblastine, does not protect the separate colchicine-binding site. It remains to be seen whether this means that the vinca alkaloids’ aggregating action is due to their dimeric character. Maytansine’s ineffectiveness as an anticancer drug in phase II clinical trials in humans, despite its great in vitro and animal in vivo potency, is remarkable. The apparent greater toxicity in humans might be related to variations in metabolism, since the chemical proved effective in animals. A comparison of maytansine metabolism in animals and humans may uncover locations in the molecule that can be changed to minimize human toxicity. The apparent greater toxicity in humans might be related to variations in metabolism, since the chemical proved effective in animals. A comparison of maytansine metabolism in animals and humans may uncover locations in the molecule that can be changed to minimize human toxicity [137,141,142,143].

### 3.14. Thapsigargin

Thapsigargin (TG) is a lactone sesquiterpene isolated from the roots of *Thapsia garganica* L. (Apiaceae) and collected from Ibiza Island [144]. Thapsigargin affects calcium homeostasis leading to increase nitric oxide concentration which results apoptosis and cell death [145]. The dysregulation of calcium ion concentration generates stress in endoplasmic reticulum leading to sequential activation of caspase. It initiates the releases apoptotic factors from the mitochondria and directs cell death (Figure 2). Thapsigargin explicitly hinders the fusion of autophagosomes with lysosomes, the last phase in the autophagic process causing cellular death [146,147]. 

**Figure 15:**
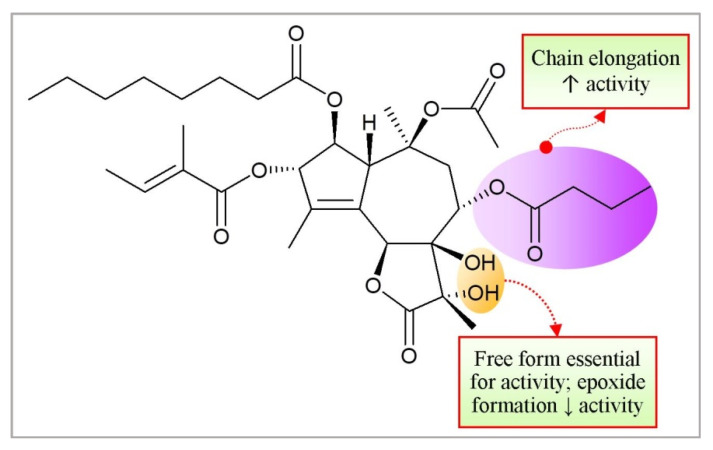
SAR of thapsigargin.

The O-2 side chain at the Tg skeleton might be mainly responsible for the inhibitory effect of Tg in SPCA1a (Figure 15). Certainly, as seen in SERCA1a, the alteration of the 7,11- gem diol in Tg into an epoxide led to an intense decline in the inhibitory effect in SPCA1a, seemingly by the loss of hydrogen donors in the molecule or by the changed conformation of the tricyclic nucleus due to the conversion of the stereochemistry at C-11. Of note, some Tg equivalents necessitate a longer pre-incubation time with SERCA1a to attain the full inhibitory potential, which can be explained by a time-dependent induced fit of Tg into the SERCA1a pocket. However, the weaker inhibitory potential of the Tg analogues in which the octanoyloxy group has been substituted with a hydrogen atom (ΔO-2), and Tg-epoxide did not significantly increase after a longer pre-incubation time with SPCA1a [148,149].

Tg promotes cancer cell death in a proliferation-independent manner in both the proliferative and quiescent stages of the cell cycle by inhibiting the SERCA pump. To determine the influence of each functional group of Tg on its effectiveness as a SERCA inhibitor, detailed structure-activity relationship experiments were conducted. Three acyloxy groups (at C-3, C-8, and C-10), the methyl group at C-4, and the lactone ring interact with the backbone of the SERCA pump via hydrophobic contacts, according to the pharmacophoric model. The octanoyloxy group’s hydrophobic chain extends into the lipid phase of a cell membrane, producing a weak contact with ATPase. The inversion of the arrangement at C-3 and C-8 proved detrimental to Tg activity [147,150,151,152].

### 3.15. Bruceantin

**Figure 16:**
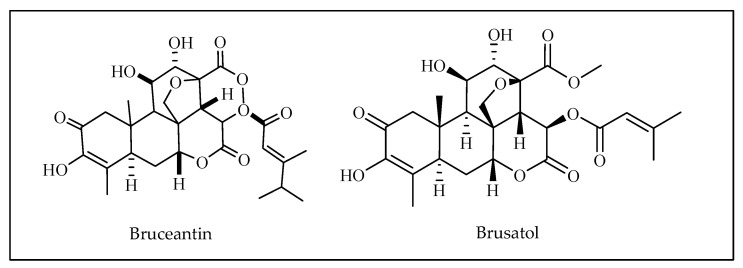
Bruceantin and brusatol.

Bruceantin (Figure 16) was first isolated from an Ethiopian plant *Brucea antidysenterica* (Simaroubaceae) and is used by local healers in the treatment of cancer. Bruceantin has been observed in the treatment of several cancers, such as B16 melanoma, colon 38, and L1210 and P388 leukaemia, in mice. The activity of bruceantin has been scientifically explicated on numerous leukaemia, lymphoma, and myeloma cell lines. However, no noticable tumor regressions were witnessed in phase I and II clinical trials, so use of this drug was dismissed. On the other hand, recommenced research proved the effectiveness of bruceantin against leukaemia, lymphoma, and myeloma cell lines in animal studies with advanced phases of the disease. It was established that treatment of HL-60 and RPMI 8226 cell lines prompted apoptosis through caspase and mitochondrial pathways. Moreover, an in vivo study on RPMI 8226 human-SCID xenografts showed that bruceantin affects early as well as advanced tumors. Bruceantin exerts its anticancer activity by inhibiting protein synthesis through interaction with peptidyltransferase [153] (Figure 2). 

Brusatol (Figure 16) and its structural correspondents were thus primed and tested for their cytotoxicity on P-388 murine leukemia cells. The replacement of the enol function at ring A with a methoxyl group showed a noticeable reduction in activity, but replacement with a hydrogen atom retained the activity. In dehydrobrusatol, the reduction was negligible, whereas in modification the decrease in activity was more noticeable as the enol group was replaced by a hydrogen atom and the 1,2-double bond was saturated. Thus, the incidence of enone-carbonyl oxygen or enolic oxygen at C-2 seems to be crucial for the compounds to exhibit the activity, but the oxygen at C-3 is not vital. Analogues formed by modification at 11-ketone analogue presented very poor activity, indicating that the replacement of AB-hydroxyl group at C-11 is essential. The 16-lactone analogue also showed borderline activity. The activity generally falls as the length of alkyl chain increases from methyl to pentyl. As a conjugate system, modification at the ring C assemblies or at the C-21 alkoxy chain lengths and by detailed assay of their effect on leukemia cells provided information on the relation between the chemical structure and the cytotoxic activity of the compounds of this series. Such knowledge should be useful for designing and synthesizing novel analogues of brusatol and bruceantin [153,154,155].

The tumor suppressive effect of brusatol has been emphasized in a number of malignancies as basic/translational research has expanded. However, there are a number of problems that prevent this chemical from being used in clinical trials. To begin with, the specificity of brusatol is unknown. While most research has identified brusatol as an NRF2 inhibitor [156], some publications have suggested alternative methods, such as direct suppression of protein synthesis [157] or c-MYC downregulation [158,159]. According to a bioinformatics analysis, brusatol could possibly target 464 proteins. Although this theory has yet to be proven in biological investigations, it suggests that off-target effects may be a problem for brusatol’s future usage. Crystallography studies clarifying the brusatol-NRF2 interaction, as well as brusatol molecular structure modification, might be viable ways to increase NRF2 targeting specificity. Second, brusatol is harmful to the entire body. In early phase clinical investigations, severe adverse effects such as hypotension, nausea, and vomiting were identified. As a result, the dose of brusatol should be carefully adjusted. Furthermore, improving brusatol specificity for NRF2 might help to decrease side effects [158]. The down-regulation of c-MYC, the activation of caspases, and the mitochondrial pathway are all required for bruceantin-induced apoptosis. These findings imply that bruceantin should be retested in a clinical environment for its efficacy against hematological malignancies in which c-MYC is produced and inhibited by the drug [159].

### 3.16. Curcumin

Curcumin is a yellow polyphenol (diferuloylmethane) derived from turmeric rhizomes (*Curcuma longa* Linn). Curcumin is from the linear diarylheptanoid group of natural compounds in which two oxy-substituted aryl groups are linked together through a seven-carbon chain. The C7 chain of this group is known to have unsaturation, oxo functions, enone moiety and a 1, 3-diketone group. Excluding the oxo and hydroxy groups, the C7 chain is generally unsubstituted. This unsaturation in the linker unit has an E-configuration. The most prevalent natural substituents are of the oxy type, such as hydroxy of methoxy elements [160,161].

**Figure 17:**
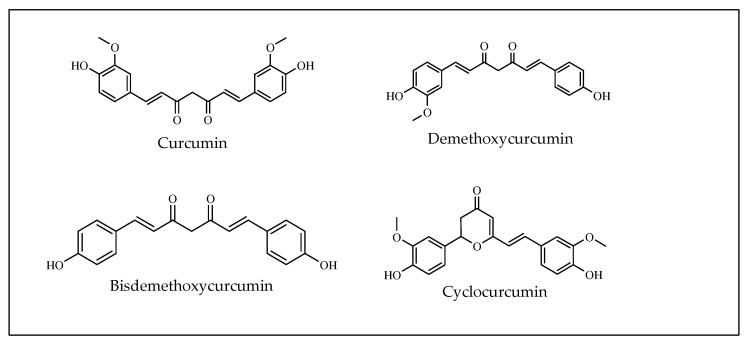
Carcuminoid analogs.

The curcuminoids (Figure 17) are well known as free radical scavengers and reactive oxygen species (ROS), such as hydroxyl radicals, superoxide radicals, singlet oxygen, peroxyl radicals and peroxynitrite, whose production is involved in the induction of oxidative stress. Recent studies suggest that curcumin is a multipotent agent that is able combat Alzheimer’s disease with the activities of scavenging radicals, obstructing amyloid-β (Aβ) accumulation and chelating metal ions [162]. Researchers have demonstrated that all of these pharmacological effects were related to the strong antioxidant effects of curcumin [163]. ROS has direct impact on the development and progression of malignancies [164]. Thus, curcuminoids can be promising candidates in the development of anticancer therapies from natural sources. 

The four chemicals have molecular weights ranging from 194 Da (ferulic acid) to 368 Da (curcumin). The molecular volume, as predicted by molecular modeling, represents the size of the molecules. The relationship between molecular volume and molecular weight has been discovered. Alog P can measure the tendency of lipophilicity in addition to retention time. The following was Alog P’s trend (however, the Alog P of the three curcuminoids was approximate): ferulic acid (1.67), curcumin (3.55), DMC (3.57), and BDMC (3.59). Following the increase in dimethoxy, the number of H-bond acceptors was reduced [165,166,167].

Widespread investigational and experimental research has symbolized that curcumin shows anticancer effects through its manifold effects on mutagenesis, cell cycle regulation, apoptosis, oncogene expression and metastasis [168]. Different phases of cancer including introduction and progression can be affected by curcumin. This compound amended histologic factors in one out of two patients with resected bladder cancer, one out of six patients with abdominal metaplasia and one out of four patients with uterine cervical intraepithelial neoplasm. In a nonrandomized open-label study, 25 patients with pancreatic cancer were enrolled in an oral curcumin administration. Among them, two patients revealed clinical responses; one had stable disease for >18 months and the other had tumor regression. Another study demonstrated that curcumin was accompanied by a significant decline in lymphocytic glutathione S-transferase (GST) activity. The GSTs are a family of phase II detoxification enzymes and have been shown to be involved in the development of resistance to chemotherapy drugs. The antitumor action of curcumin is mediated via its anti-proliferative effect on multiple cancers, inhibitory effect on transcription factors and downstream gene products, and modulatory effect on growth factor receptors and cell adhesion molecules involved in angiogenesis, tumor growth and metastasis [169,170,171].

## 4. Current Drug Therapy vs. Bioactive Phytochemicals: Limitations and Challenges

Many studies have been carried out to try and decrease the dangerous unwanted side effects of drugs in most cancer therapy procedures, such as anticipating the side effects on the adjacent cells and tissues, expanding medicate collection and adequacy within the injury, creating novel medicate conveyance and focusing on frameworks. There are many strategies that aim to cure cancers, for instance, surgical operation on tumors, chemotherapy, cancer vaccinations, photodynamic therapy, radiotherapy, immunotherapy, stem cell alteration, or a combination thereof. These are regularly observed to have extreme side effects. Such outcomes include constrained bioavailability, toxicity, non-specificity, rapid clearance, and limitation in metastasis [172,173]. Treatment strategies of cancer rely on the cancer’s type, stage and place. Chemotherapeutic operators include cytostatic and cytotoxic drugs that appear to be life-saving strategies alone or in combination with other cancer treatments. These chemotherapeutic agents contain topoisomerase inhibitors, e.g., irinotecan (side effects include: neutropenia, sensory neuropathy, and diarrhoea); cyclophosphamide (undesired effects include: gastrointestinal toxicity, cardiovascular toxicity, pulmonary, nephrotoxicity and hematologictoxicity, and doxorubicin (side effects include cardiotoxicity); microtubules acting agent, e.g., vincristine, vinblastine, paclitaxel and docetaxel; and alkylating agents, e.g., oxaliplatin, carboplatin, melphalan, and cisplatin. These drugs are extraordinarily powerful in treating an extensive variety of cancers; however, these pills also have some disadvantages (side effects, cost, exceptional complexity, environmentally ufriendly, and harmful). Some cells in our frame multiply swiftly under ordinary physiological situations such as hair follicle cells, digestive tract cells and bone marrow cells, to name a few. This shows that anticancer pills additionally affect dividing ordinary cells. This is a massive undertaking and cause dangerous side effects. These side effects may include diminished blood generation, immunosuppression, hair falls, coronary heart diseases, GIT aggravation and apprehensive clutters. Another obstacle is that most cancers cells resist remedies as they mutate; e.g., genes resistant to drug (abca4 and abca12) were over-expressed in most human mcf-7 breast cancer cells when docetaxel was administered. In any case, while phytochemical curcumin was applied in affiliation with docetaxel, overexpression of drug resistant genes were chnaged. Moreover, drug resistance to existing cancer treatments, which leads to the rapid buildup of CSCs, disease recurrence in treated tumors, and extensive metastasis in the late stages of cancer, necessitates fresh research into effective strategies to alleviate these burdens. To totally eliminate drug-resistant cancer cells and CSCs, novel combination treatments targeting multiple elements of the tumor microenvironment are needed. According to a new study on zebrafish embryos, silver nanoparticle toxicity is linked to accessible silver ions. Modern research demonstrated undetectable toxicity in healthy volunteers after the oral ingestion of commercially available silver nanoparticles. The biodegradability of SNPs is another important consideration. The renal clearance of glutathione-coated luminous gold nanoparticles was also recorded. According to a recent study, biogenic SNPs are generally less cyto-/genotoxic in vivo than chemically generated SNPs [26,174,175,176,177,178].

A number of natural phytochemicals have been reported as having significant anticancer properties and many of them have been investigated under clinical trials (Table 2). The development of these bioactive compounds is a complex and time-consuming process. The complexity of the extraction, purification, isolation and characterization of the natural compounds may be reduced by advanced analytical and computational technologies which enhance the treatment cost and result in an extra burden to patients. Furthermore, adequate biopharmaceutical and clinical evidence is essential for delivering these biocompounds from the laboratory to the patient. Many of them are proven to besafe, therapeutically effective, and biocompatible in clinical trials and are thus used in cancer treatments. Numerous challenging factors have created limitations in the development of natural anticancer biomolecules as drug products. Along with toxic side effects, lower water solubility, decreased absorption, lack of selectivity to targeted cancer cells, and sub-therapeutic activity are the major obstacles for anticancer drug development from natural sources. Potent therapeutic compounds such as colchicine, camptothecin, and podophyllotoxin showed severe side effects which limit their uses [179]. The cytotoxicity of natural phytocompounds and their selectivity to cancer cells can be improved using extensive SAR analysis. For example, along with cytotoxicity, the selectivity of triterpene natural products to A431 and C6 glioma cell lines was found to be enhanced by the trivial modification of functional group substitutions as well as their stereochemistry [6].

In the modern day, novel drug delivery systems (including nanotherapeutics) are utilized extensively to minimize solubility and penetration issues. Moreover, the specificity and selectivity of the biomolecules have been enhanced by nanotechnology using active and passive targeting approaches [5]. Apart from these, nanotherapeutics has enhanced the bioavailability of natural molecules in cancer therapy. Thus, the dose and exposure of the cytotoxic biomolecules has been reduced, resulting in the minimization of toxic effects. For example, curcumin delivered by hyaluronic acid-conjugated silica nanoparticles showed enhanced anticancer activity along with enabling higher bioavailability [187].

## 5. Conclusions

For centuries, plants have been utilized extensively as medicinal agents in order to treat a wide assortment of infections. Over the ages, human beings have relied on conventional home-grown herbal agents to fulfill their health prerequisites. These plant-derived natural resources have proved to be non-toxic and are potential modes of cancer management and therapy. Bioactive phytocompounds such as VA, taxanes, ellipticine, camptothecin, combretastatin, curcumin, podophyllotoxin, homoharringtonine and others, are acknowledged for their potential anticancer effects on various neoplastic diseases. SAR studies demonstrate that potent analogs possess multiple effects on numerous molecular targets of malignant cells. These analogs can be developed as non-toxic and therapeutically effective drug products to combat various malignancies. 

Considering drug development-related challenges such as physicochemical properties, selectivity and specificity, pharmacokinetic behaviors, and clinical safety issues, advanced analytical (as well as in silico and computational technologies) should be adopted. Moreover, nanotechnology along with site-directed drug delivery approaches has raised hopes for the non-toxic and successful development of anticancer therapy from natural sources. Extensive research in this field is critical in order to cope with challenges such as the increasing rate of malignancy, and the concurrent mortality and morbidity of human beings.

## Figures and Tables

**Table 1:** Vinca alkaloids (VAs) and their applications in different treatments for cancers along with their most common side effects.

VAs	Cancers	Common Side Effects
VBL	Leukemia, non-Hodgkin’s and Hodgkin’s lymphoma, breast cancers, nephroblastoma, Ewing’s sarcoma, small cell lung cancer, testicular carcinoma, and germ cell tumors	Toxicity to white blood cells, nausea, vomiting, constipation, dyspnea, chest or tumor pain, wheezing, fever, antidiuretic hormone secretion, and weight loss
VCR	Philadelphia chromosome-negative acute lymphoblastic leukemia, B-cell lymphoma, metastatic melanoma, breast cancer, glioma, colorectal cancer, non-Hodgkin’s and Hodgkin’s lymphoma, neuroblastoma, rhabdomyosarcoma, multipole myeloma, and Wilm’s tumor	Important peripheral neuropathy, nausea, vomiting, diarrhea, bloating, stomach/abdominal cramps, mouth sore, dizziness, headache, hair loss, constipation, loss of appetite, and weight loss
VDS	Pediatric solid tumors, malignant melanoma, blast crisis of chronic myeloid leukemia, acute lymphocytic leukemia, metastatic colorectal cancer, breast cancer, and renal and esophageal carcinomas	Leukopenia, thrombocytopenia, fatigue, constipation, sore mouth, difficulty swallowing paralytic ileus, loss of sensation, nerve pain, diarrhea, convulsions, depression, and weight loss
VRL	The wide antitumor spectrum of activity, such as advanced breast cancer, advanced metastatic non-small cell lung cancer, and rhabdomyosarcoma	Neuropathy, nausea or vomiting, muscle weakness, constipation, diarrhea, anemia, and weight loss
VFN	Metastatic and advanced urothelial cancer after failure of platin containing therapy	Neuropathy, nausea or vomiting, muscle weakness, constipation, abdominal pain, vomiting or nausea, stomatitis, diarrhea, alopecia myalgia, fatigue, and weight loss

**Table 2:** Plant derived drugs investigated under clinical trials.

Class of Phytochemicals	Pharmacological Action	Clinical Trial	Type of Cancer	Molecular Targets	Ref.
Camptothecin Irinotecan Topotecan	Stabilizes topoisomerase I-DNA complex thereby preventing re-ligation of single strand breaks resulting in lethal double-stranded breaks in DNA.	Prospective phase I clinical trial	Ovarian, cervical, colorectal, and small cell lung cancer (SCLC)	Topoisomerase I	[180]
Combretastatin A4	Inhibits polymerization of tubulin causing disruption of the tumor endothelial cells lining the tumor vasculature	Prospective phase I clinical trial	Polypoidal choroidal vasculopathy, anaplastic thyroid cancers	Tubulin	[181]
**Curcumin**	Multiple actions on mutagenesis, cell cycle regulation, apoptosis, oncogene expression and metastasis	Prospective phase II clinical trial	Patients with advanced pancreatic cancer, urinary bladder cancer, uterine cervical neoplasm, or intestinal metaplasia	-	[182]
**Homoharringtonine**	Binds to large ribosomal subunit, which affects chain elongation and prevents protein synthesis	Prospective phase I/II clinical trial	Chronic myeloid leukemia	Ribosomoal protein	[183]
**Podophyllotoxin** Etoposide Teniposide	Inhibits DNA synthesis by forming a complex with topoisomerase II and DNA.	Prospective phase I clinical trial	Osteosarcoma, NSCLC cervical, nasopharyngeal, colon, breast, prostate, and testicular cancer	Topoisomerase II	[184]
**Taxanes** Cabazitaxel Docetaxe Paclitaxel	Inhibit microtubule function resulting in cell cycle arrest and aberrant mitosis.	Phase II clinical trial	NSCLC, head and neck, breast, prostate, gastric adenocarcinoma	Tubulin	[185]
**Vinca alkaloids** Vinblastine Vincristine Vindesine Vinflunine Vinorelbine	Inhibit microtubule polymerization and assembly, leading to metaphase arrest and cell death.	Phase II clinical trial	NSCLC, breast, lung, leukemia, Hodgkin and non-Hodgkin lymphomas, testicular carcinoma, Kaposi’s sarcoma, and second-line transitional cell carcinoma of the urothelium (TCCU)	Tubulin	[186]

## Data Availability

The data presented in this study are available on request from the corresponding author.
